# Parotid Gland Tumors: An Institutional 8-Year Retrospective Study Spanning the COVID-19 Pandemic and Global Diagnostic Trends

**DOI:** 10.3390/jcm14207382

**Published:** 2025-10-19

**Authors:** Eduard Gidea-Paraschivescu, Ruxandra Elena Luca, Cristian Adrian Ratiu, Ciprian Ioan Roi

**Affiliations:** 1 Emergency University Municipal Hospital Timisoara—Oral and Maxillofacial Surgery Clinic, Take Ionescu Bd., No. 5, 300062 Timișoara, Romania; eduardparaschivescu@gmail.com; 2 University Clinic of Oral Rehabilitation and Dental Emergencies, Faculty of Dentistry, “Victor Babes” University of Medicine and Pharmacy, Eftimie Murgu Square No. 2, 300041 Timișoara, Romania; 3 Interdisciplinary Research Center for Dental Medical Research, Lasers and Innovative Technologies, Revolutiei 1989 Avenue No. 9, 300070 Timișoara, Romania; 4 Faculty of Medicine and Pharmacy, University of Oradea, 1st December Square 10, 410073 Oradea, Romania; 5 Department of Anesthesiology and Oral Surgery, Research Center of Dento-Alveolar Surgery, Anesthesia and Sedation in Dental Medicine, “Victor Babes” University of Medicine and Pharmacy, Eftimie Murgu Square No. 2, 300041 Timișoara, Romania; ciprian.roi@umft.ro

**Keywords:** parotid tumor, salivary glands, head and neck neoplasms, salivary gland neoplasms, pandemic

## Abstract

**Background/Objectives**: Despite the relative rarity of salivary gland tumours (SGT), they are a complex and challenging pathology. This is primarily due to the complexity of surgical treatment, the difficulty of diagnosis, and the sometimes ambiguous prognosis. **Methods:** This retrospective study examined parotid gland tumors in patients admitted for diagnosis and treatment at the Municipal Hospital of Timisoara–Oral and Maxillofacial Surgery Clinic, Romania, from 2016 to 2023, with the objective of verifying the hypothesis regarding the increasing incidence of benign tumors in the major salivary glands, particularly the parotid gland. **Results**: A total of 207 consecutive parotid nodular lesion cases were analysed, with 186 having a histopathological analysis. The findings encompass demographic patterns, temporal dynamics, histopathological profiles, malignancy characteristics, and statistical associations. The cohort was evenly distributed by sex (102 females, 105 males) with a median age of 58 years (IQR: 46–69). The largest age group was ≥60 years (n = 99; 47.8%), followed by 40–59 years (n = 76; 36.7%) and <40 years (n = 32; 15.5%). No significant sex difference in age distribution was observed. Annual case volumes showed a high plateau between 2017–2019 (40–41 cases/year), then fell sharply during the pandemic (2020–2022), reaching a nadir in 2021 (11 cases). A partial rebound occurred in 2023 (21 cases). The relative proportion of malignant diagnoses remained stable between pre-pandemic (20.0%) and pandemic/post-pandemic (8.9%) intervals, consistent with prioritization of oncologic surgeries during service restrictions. Benign tumors predominated (n = 126; 60.9%), led by pleomorphic adenoma (n = 64; 50.8% of benign) and Warthin tumor (n = 59; 46.8% of benign). Malignant tumors accounted for 31 cases (15.0%), most commonly squamous cell carcinoma (n = 6), mucoepidermoid carcinoma (n = 6), and adenocarcinoma of salivary origin (n = 5). Mann–Whitney U tests confirmed no significant differences in median age between malignant and benign cases, or between pre-pandemic and pandemic/post-pandemic intervals. Odds ratios suggested clinically relevant but non-significant increases in malignancy risk for males (OR ≈ 2.1) and for patients ≥60 years (OR ≈ 1.2). Linear regression of annual case counts revealed a downward slope of –3.5 cases/year (*p* ≈ 0.074), driven by the sharp pandemic-era decline. **Conclusions**: This study illustrates that, despite a significant decrease in surgical case volume during the COVID-19 pandemic, the relative distribution of parotid tumor pathology remained stable. Malignant lesions mostly occurred in older patients and males, with no statistically significant differences seen among demographic or clinical subgroups. The preservation of consistent malignancy detection rates, despite limited surgical capacity, underscores the efficacy of oncologic prioritization under healthcare disruptions.

## 1. Introduction

Major and minor salivary glands are exocrine glands responsible for the production, modification, and secretion of saliva into the oral cavity [1]. They are categorized into three paired major salivary glands and 600–1000 minor salivary glands corresponding to the oral cavity and oropharyngeal mucosa [1,2,3].

Salivary gland tumors (SGT) are considered relatively rare, the percentage varying between 3–10% of all head and neck neoplasms, with an annual estimated global incidence ranging from 0.4 to 13.5 cases per 100,000 individuals, of which, only 10–20% are malignant type [4,5,6,7,8,9]. Among these, parotid tumors are considered a heterogeneous group, accounting for 85% of all salivary gland tumors, 2–6% of the head-neck tumors and about 1% of all tumors [10].

Studies conducted so far showed us that the prevalent age for these neoplasms is the 6th decade, with benign SGT usually appearing earlier, between the 4th and the 5th decade of life [11]. It appears that the benign SGT occur more frequent in females, whereas malignant tumors show equal distribution amongst gender. The most common salivary glands to be involved in this pathology include parotid gland (80%) followed by submandibular gland (10–15%), sublingual and minor salivary glands [3,12,13].

In the specialized literature we can find the so-called rule of 80 referring to the parotid gland pathology: 80% of parotid tumors are benign, of this 80% are pleomorphic adenomas (mixed benign tumors) and 80% located in the superficial (lateral) lobes [14]. The pleomorphic adenoma is the most common tumor of the salivary glands with a reported incidence of 2.4 to 3.05/100,000 persons/year. Although a common disease, demographic data are scarce and many studies analyze only small or selected populations [15,16]. Except for pleomorphic adenoma, the World Health Organization (WHO) Classification of Head and Neck Tumors includes another 38 different histological subtypes of SGT, including Warthin tumor, mucoepidermoid carcinoma, and adenoid cystic carcinoma [1,17].

SGTs represent a challenging and complex pathology, mainly due to the difficulty of diagnosis, the complexity of surgical treatment and the sometimes-uncertain prognosis. Etiologic factors of this group of neoplasms are poorly understood. There are studies that discuss the following possible causal or favoring factors: the ionizing radiation, sunlight, chemotherapy, smoking, vitamin A deficiency, high consumption of processed meat, low-vegetable and high-animal fat diets, obesity, Epstein–Barr virus, immunodeficiency, HIV infection [1,18,19,20]. What is common to many studies is the conclusion that this type of SGTs have inconsistent characteristics in different countries, therefore the clinico-pathologic profile seems to be influenced by the geographic location and ethnic factors [19,21].

The salivary glands, particularly the parotid gland, play a crucial role in the pathogenesis of COVID-19 and the entry of the virus into the body. Recent studies [22] demonstrate that components of the plasminogen (Plg)/plasmin (Pm) system are present in oral cavity tissues, including the salivary gland, and play a role in microbial infection and inflammation. The Plg/Pm system serves two primary functions: the degradation of fibrin deposits in the bloodstream or damaged tissues, known as fibrinolysis, and non-fibrinolytic actions that involve the proteolytic modulation of proteins. Both functions can be observed during inflammation. The virus responsible for coronavirus disease 2019 (COVID-19) utilises both the fibrinolytic and non-fibrinolytic functions of the Plg/Pm system within the oral cavity. During COVID-19, established coagulopathy accompanied by microthrombi necessitates continuous activation of the fibrinolytic function. Viral entry is influenced by receptors like TMPRSS2, essential in the oral cavity, resulting in an altered immune response characterised by a peak in cytokine storm syndrome. Salivary gland cells, particularly ductal epithelial and serous acinar cells, exhibit high expression levels of the angiotensin-converting enzyme 2 (ACE2) receptor and TMPRSS proteases, which are essential for the entry of SARS-CoV-2. Additionally, plasmin plays a role in the cleavage of the S protein of SARS-CoV-2, facilitating its entry. Viruses like SARS-CoV-2 utilise the fibrinolytic system to facilitate infection of host cells [22].

In this context, the aim of our study was to verify the hypothesis whether the upward trend in the incidence of tumors applies to the parotid salivary glands admitted in our unit. We were interested in the predominant histopathological type of tumors in the parotid glands, as well as any correlations between the incidence of tumors and gender, patient age, vicious habits, and other medical history that could influence SGTs.

## 2. Materials and Methods

Our retrospective study focused on the analysis of parotid gland tumors in patients who were admitted for diagnosis and treatment in the Municipal Hospital of Timisoara- Oral and Maxillofacial Surgery Clinic, Romania between 2016–2023 and aimed to verify the hypothesis whether the upward trend in the incidence of benign tumors applies to the major salivary glands, especially the parotid gland. The study was performed at the Municipal Hospital of Timisoara-Oral and Maxillofacial Surgery Clinic, Romania, affiliated with the Victor Babes University of Medicine and Pharmacy in Timisoara. The Oral and Maxillofacial Surgery Clinic of Timișoara is a longstanding medical facility that has been serving Timișoara, Timiș County, and adjacent counties, catering to a population of over 1.5 million residents throughout nearly five counties for over 60 years. The study protocol was approved by the Research Ethics Committee of “Victor Babes” University of Medicine and Pharmacy in Timisoara, Romania (approval no. 16/08.03.2024).

We retrieved all records from the hospital database Electronic Health Record (EHR InfoWorld Connecting Healthcare), of patients presenting salivary glands nodular tumor formations, who were discharged between 1 January 2016 00:00 and 31 December 2023 23:59, totaling in the initial phase, a number of 688 patients. A total of 207 patients were identified after removing non-tumorous formations, and their anatomo-pathological studies were reviewed to confirm the conclusive diagnoses.

All the cases in this study adhered to the Oral and Maxillofacial Surgery Clinic protocol for managing parotid nodular formations, which included preoperative imaging investigations (CT, IRM, ultrasound and/or orthopantomography, to exclude a bone lesion that has extended into soft tissue, simulating a parotid lesion) and FNA/excisional biopsy, subsequent excision of the tumor, and, in instances of malignancy, lateral-cervical lymph node dissection, followed by radiotherapy and chemotherapy. During pandemic, all individuals admitted to the maxillofacial surgery clinic during the pandemic underwent testing. Those identified as COVID-positive were referred to specialised departments and later returned for treatment of their condition within our area of focus. No cases admitted to the maxillofacial surgery clinic were treated following a positive test result.

Out of the 207 cases examined, 21 specimens were deemed uncertain due to insufficient viable tissue or equivocal histological results. These patients were excluded from further malignancy pattern analysis, resulting in 186 analysable instances. Consequently, we provide distributions and subgroup analyses exclusively based on this analysable cohort.

The findings integrate demographic patterns (distribution by sex and age groups), temporal dynamics, histopathological profiles, malignancy characteristics (benign, malign, non-neoplastic lesions), and statistical associations.

## 3. Results

### 3.1. Demographic Characteristics

The analytic cohort comprised 207 patients. The mean age at presentation was 56.5 ± 14.6 years (median 59.0; interquartile range 46.0–67.5; range 16–90 years), indicating a distribution skewed toward older adults. Sex distribution was essentially balanced—105/207 (50.7%) male and 102/207 (49.3%) female—without an overall sex predominance across the study period. By age group, <40 years accounted for 32/207 (15.5%), 40–59 years for 76/207 (36.7%), and ≥60 years for 99/207 (47.8%), showing that almost half of the cases occurred in the elderly.

**Figure 1:**
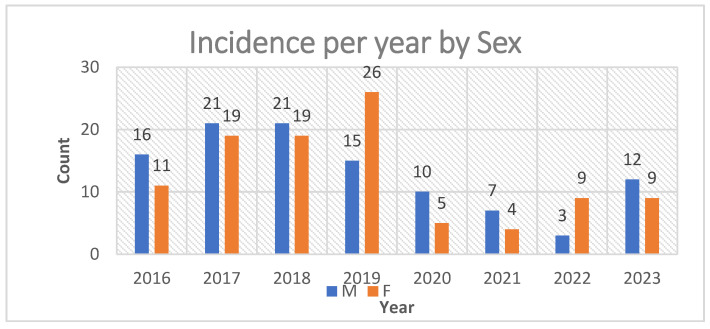
Incidence per year by Sex.

**Figure 2:**
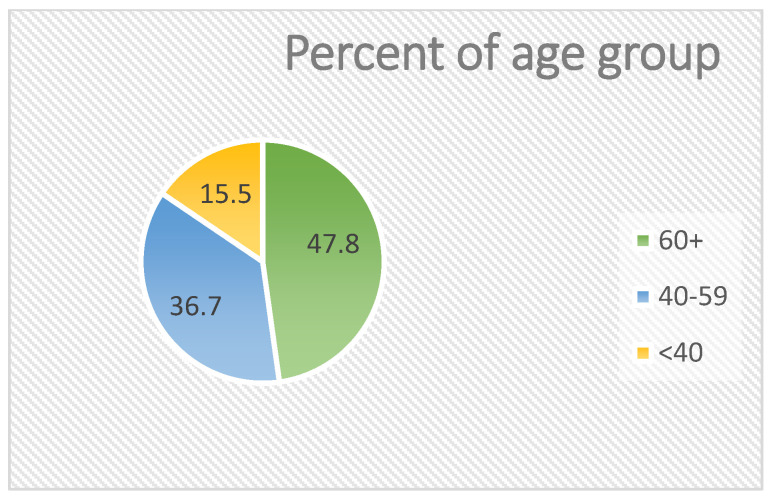
Distribution of patients by age group.

**Figure 3:**
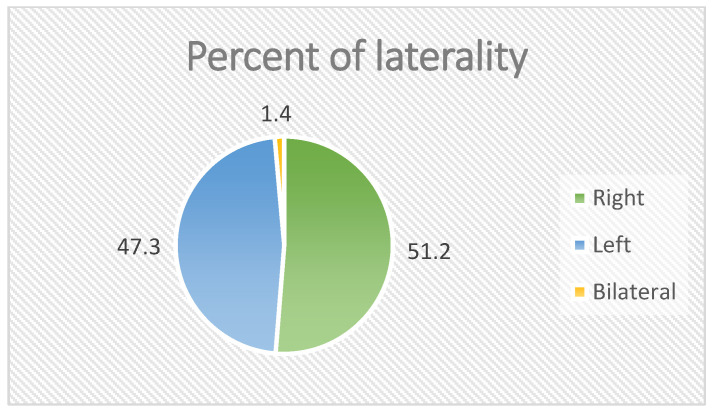
Laterality distribution of patients.

Year-by-year inspection of sex-specific incidence suggests relative balance between males and females across most years, with a notable female peak in 2019 (see Figure 1). For clarity in the remainder of the manuscript, we refer to three age bands (<40, 40–59, ≥60 years) when stratifying subsequent outcomes. Baseline distributions by sex, age band, and laterality are summarized in Table 1, and visualized in Figure 2 and Figure 3.

### 3.2. Temporal Trends

**Figure 4:**
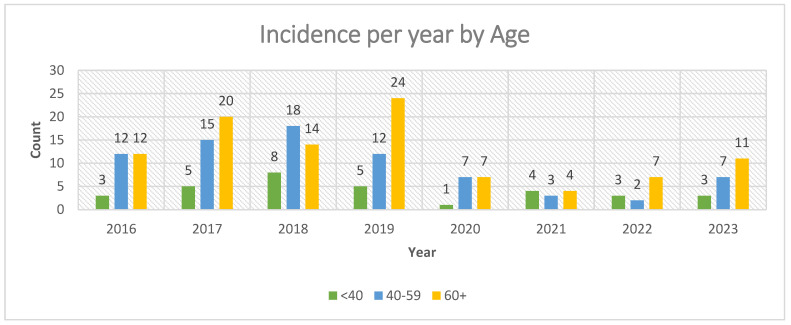
Incidence per year by age.

When analyzed by year (Figure 4), case numbers showed a bimodal distribution with two major peaks, 2017–2019, followed by a reduced incidence coinciding with the COVID-19 pandemic years (2020–2022), and a partial rebound in 2023. The highest annual incidence is recorded in 2019, with 41 cases (19.8%), while the lowest incidence occurred in 2022, with 12 cases (5.8%).

**Figure 5:**
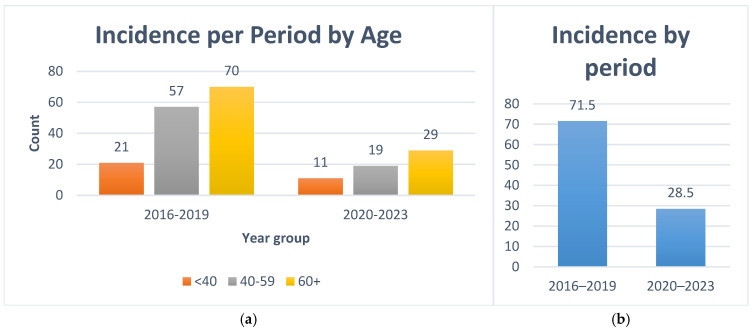
(**a**)–Incidence per Period by Age, (**b**)–total incidence per period.

To explore the potential influence of the COVID-19 pandemic and associated restrictions on medical services, the cohort was divided into two periods. First period, pre-pandemic (2016–2019) has 148/207 cases (71.5%) and pandemic and post-pandemic recovery (2020–2023) has 59/207 cases (28.5%), as can be seen in Figure 5.

This represents a 60.1% decrease in case volume between the two periods. The reduction was particularly evident in elective benign tumor resections, while the proportion of malignant tumors (discussed in Section 3.4) did not decline proportionally, suggesting that urgent oncologic surgeries continued despite restrictions.

The decline in case numbers during 2020–2022 is likely multifactorial, but a major contributing factor was the COVID-19 pandemic-related restriction of non-urgent surgical activity, limited patient access to specialty clinics, and patient hesitancy to seek medical attention for non-life-threatening conditions. The partial rebound in 2023 may reflect a resumption of deferred surgical cases and increased patient presentation following the lifting of pandemic measures.

Figure 5a illustrates the annual incidence by age group, demonstrating that all age categories were affected by the decline, with the most pronounced drop in patients ≥60 years, potentially due to higher vulnerability and healthcare avoidance during the pandemic. Table 2 presents the detailed comparison between the pre-pandemic and pandemic/post-pandemic periods, including absolute numbers and percentages for each age group and sex.

### 3.3. Histopathological Diagnosis

**Figure 6:**
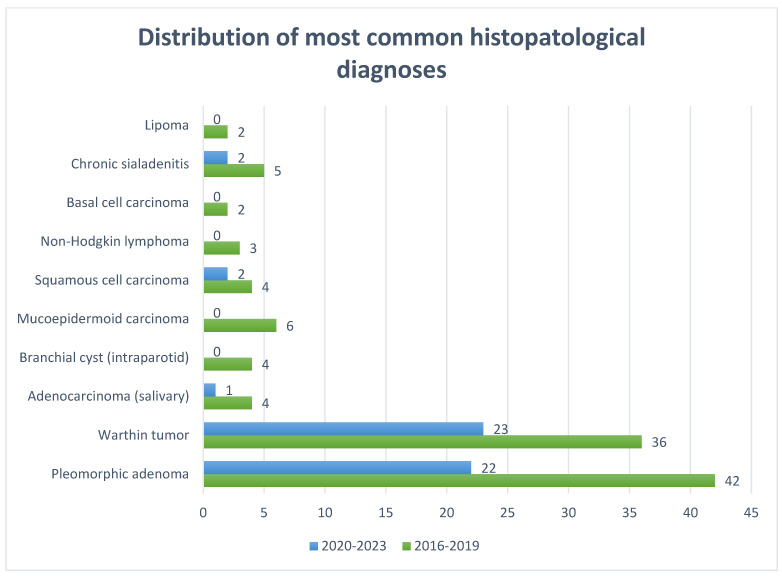
Distribution of the most common histopathological diagnoses in the study cohort.

Histopathological evaluation of the 207 parotid nodular lesions identified 27 distinct diagnostic entities. These were grouped into benign epithelial tumors, malignant epithelial tumors, lymphoid neoplasms, non-neoplastic lesions, and other rare cases. Table 3 summarizes the absolute counts for each diagnosis in the pre-pandemic (2016–2019) and pandemic/post-pandemic (2020–2023) periods while in Figure 6 a bar chart representing the distribution of most common histopathologic diagnoses.

Benign epithelial tumors dominated the series, accounting for over half of all cases. Pleomorphic adenoma was the most common diagnosis (64/207; 30.9%), with 42 cases pre-pandemic and 22 cases during the pandemic/post-pandemic interval. Warthin tumor ranked second (59/207; 28.5%), with a similar temporal distribution (36 vs. 23 cases). Other benign tumors were rare, including lipoma (n = 2) and oncocytic cyst (n = 1). Malignant epithelial tumors accounted for a relevant minority of cases. The most frequent were squamous cell carcinoma (6/207; 2.9%), adenocarcinoma of salivary origin (5/207; 2.4%), mucoepidermoid carcinoma (6/207; 2.9%), and malignant epithelial carcinoma (NOS) (4/207; 1.9%).

Non-neoplastic lesions were less frequent but clinically relevant: chronic sialadenitis (n = 7), reactive lymph node (n = 4), branchial cyst (n = 4), salivary duct cyst (n = 2), acute suppurative sialadenitis (n = 1), benign lymphoepithelial lesion (n = 1), salivary cyst (n = 1), epidermoid cyst (n = 1), and normal adipose tissue (n = 1). Rare inflammatory lesions included desmoid-type fibromatosis (n = 1).

Of the 207 cases reviewed, 21 specimens were classified as indeterminate due to lack of viable tissue or inconclusive histopathological findings. These cases were excluded from further malignancy pattern analysis, leaving 186 analyzable cases. Therefore, the following sections (3.4 onward) report distributions and subgroup analyses based solely on this analyzable cohort.

The temporal distribution demonstrated that several uncommon entities (e.g., mucoepidermoid carcinoma, branchial cysts, non-Hodgkin lymphoma) were not observed in the 2020–2023 interval, reflecting reduced elective surgical activity and stricter case selection during the pandemic.

### 3.4. Malignancy Patterns

**Figure 7:**
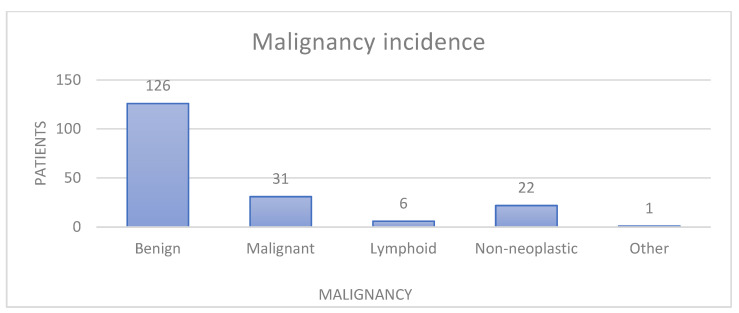
Malignancy incidence on total patients.

Histopathological evaluation was performed for all 207 parotid nodular lesions. Of these, 21 cases remained indeterminate and were excluded from the classification analysis. The final evaluable cohort therefore comprised 186 cases. Histopathological classification of the 186 parotid lesions showed that the majority were benign tumors (126 cases; 67.74%), followed by malignant tumors (31 cases; 16.66%), non-neoplastic lesions (22 cases; 11.82%), lymphoid neoplasms (6 cases; 3.22%), and other rare stromal lesions (1 case; 0.53%) (Figure 7).

Benign tumors were dominated by pleomorphic adenoma and Warthin tumor, which together accounted for nearly 60% of the series.

Malignant cases included both primary salivary gland carcinomas (e.g., squamous cell carcinoma, mucoepidermoid carcinoma, adenocarcinoma, acinic cell carcinoma, myoepithelial carcinoma) and rare variants such as oncocytic carcinoma, basal cell carcinoma, sarcoma, and cribriform adenocarcinoma. A single case of metastatic melanoma to the parotid lymph node was also identified.

The overall incidence of malignancy (15.0%) is in line with epidemiological studies from tertiary referral centers, reinforcing the predominance of benign pathology in surgically excised parotid nodular lesions.

#### 3.4.1. Malignancy by Period

When divided into pre-pandemic (2016–2019) and pandemic/post-pandemic (2020–2023) intervals, a clear decline in malignant parotid tumors was observed. In 2016–2019, 26 malignancies were identified among 130 analyzable cases, corresponding to a malignancy rate of 20.0%. By contrast, in 2020–2023, only 5 malignancies were recorded among 56 analyzable cases, yielding a malignancy rate of 8.9%. This represents a relative reduction of approximately 55% and an absolute difference of 11.1 percentage points between the two periods (Table 4).

**Figure 8:**
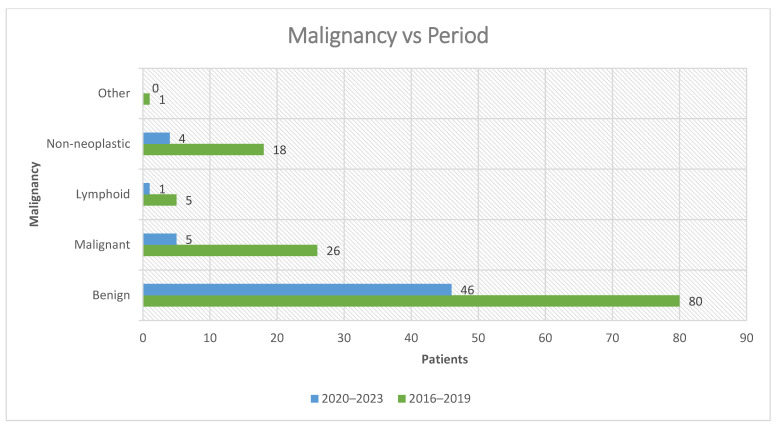
Distribution of the most common malignancy in the study cohort.

Benign tumors dominated both intervals (80 cases in 2016–2019 vs. 46 cases in 2020–2023). Lymphoid neoplasms were less frequent but present in both intervals (5 vs. 1), whereas non-neoplastic lesions showed a similar decline (18 vs. 4). Only a single case from the “other” category (desmoid-type fibromatosis) was observed in 2016–2019, with none in 2020–2023 (Figure 8).

These findings underline that, despite prioritization of oncologic and symptomatic cases during COVID-19 restrictions, the absolute number and proportion of malignant tumors decreased in the pandemic/post-pandemic interval. This likely reflects reduced patient access to healthcare, delays in diagnostic workup, and deferral of non-urgent surgical interventions, with benign tumors being most affected by postponement.

#### 3.4.2. Malignancy by Age Group

**Figure 9:**
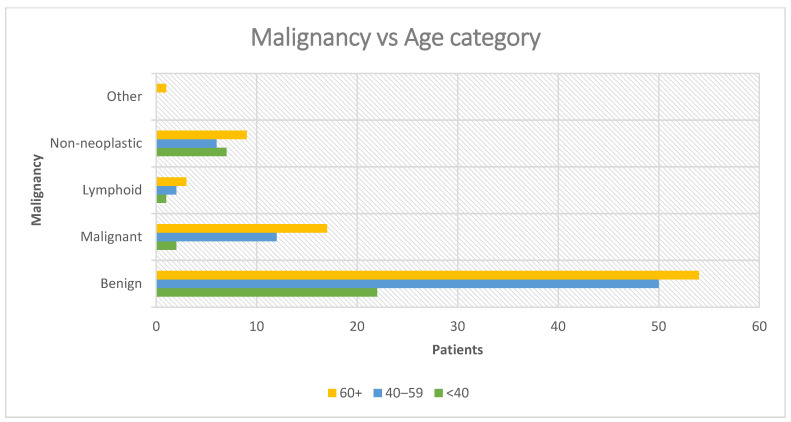
Incidence of malignancy by age group.

An age-dependent gradient in malignancy incidence was observed and it is presented in Table 5 and Figure 9. This pattern suggests a clear age-associated risk, consistent with previous epidemiological studies indicating that the probability of parotid malignancy increases with age, likely due to cumulative genetic alterations and prolonged exposure to environmental risk factors.

Benign tumors remained the most frequent category across all age groups, particularly in patients younger than 60 years. Non-neoplastic lesions were relatively more common in the <40-year group, while “Other” diagnoses, including rare entities such as sarcoma and desmoid-type fibromatosis, were almost exclusively encountered in the ≥60-year group.

#### 3.4.3. Malignancy by Sex

**Figure 10:**
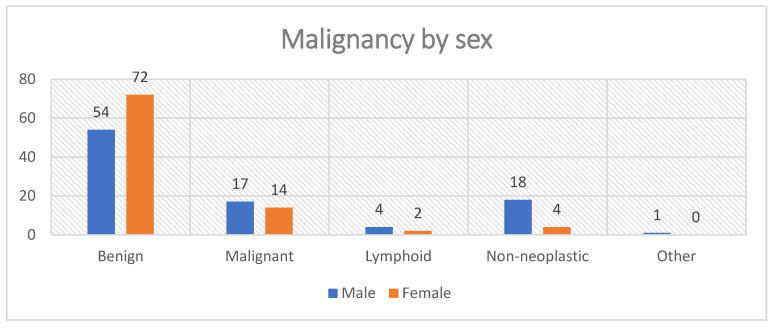
Distribution of histopathological categories by sex.

When stratified by sex, male patients had a higher proportion of malignant tumors than females (17/94; 18.1% vs. 14/92; 15.2%). Benign tumors were predominant in both groups—54/94 (57.4%) in males and 72/92 (78.3%) in females. Figure 10 illustrates these distributions graphically, highlighting the predominance of benign pathology in both sexes, the higher relative burden of malignant and non-neoplastic lesions in males, and the clear female predominance for benign tumors.

#### 3.4.4. Histological Spectrum of Malignant Cases

From the total cases analyzed, 31 (15.0%) were confirmed as malignant tumors, comprising both primary salivary gland cancers and metastatic lesions.

The most common histological type was squamous cell carcinoma (n = 6; 19.4%), followed by mucoepidermoid carcinoma (n = 6; 19.4%), and adenocarcinoma of salivary origin (n = 5; 16.1%). Other frequent diagnoses included lymphoma (n = 6; 19.4%) and malignant epithelial carcinoma, NOS (n = 4; 12.9%). Less common entities such as acinic cell carcinoma (n = 2), myoepithelial carcinoma (n = 2), basal cell adenocarcinoma (n = 2), oncocytic carcinoma (n = 1), sarcoma (pleomorphic) (n = 1), cribriform adenocarcinoma of the salivary gland (n = 1), and metastatic melanoma to the parotid lymph node (n = 1) were rare but clinically relevant (Table 6).

A slight predominance in males was observed (19/31; 61.3%) compared with females (12/31; 38.7%).

When stratified by age, malignant tumors were uncommon in patients <40 years (2/32; 6.3%), increased in those aged 40–59 years (12/70; 17.1%), and were most frequent in patients ≥60 years (17/84; 20.2%).

This distribution reinforces the association between increasing age and the risk of malignant transformation in parotid tumors, aligning with trends reported in previous epidemiological studies.

#### 3.4.5. Histological Spectrum of Benign Cases

Among the analyzed parotid nodular lesions, 126 (60.9%) were classified as benign tumors. The distribution was dominated by two major entities: pleomorphic adenoma (n = 64; 50.8%) and Warthin tumor (n = 59; 46.8%), which together accounted for nearly all benign cases. Other rare benign lesions included lipoma (n = 2; 1.6%) and oncocytic cyst (n = 1; 0.8%).

A modest female predominance was observed (72/126; 57.1% females vs. 54/126; 42.9% males). In terms of age distribution, benign tumors were most frequent in patients <60 years, with 72/102 (70.6%) cases in this group. Pleomorphic adenoma was more common in younger adults, particularly in the <40 and 40–59 age groups, while Warthin tumor predominated in older adults (≥60 years).

This age-related divergence reflects known biological patterns: pleomorphic adenoma typically affects younger and middle-aged adults, while Warthin tumor is strongly associated with older age and smoking habits. Rare benign entities (lipoma, oncocytic cyst) were confined to patients aged ≥60 years (Table 7).

### 3.5. Statistical Analysis

All statistical analyses were conducted using a two-sided significance threshold of α = 0.05.

For categorical variables (e.g., sex, laterality, diagnostic category, period), we evaluated associations using the chi-square (χ^2^) test of independence, which is designed to test whether two categorical variables are statistically related. The chi-square test compares the observed frequencies in each category with the frequencies expected if the two variables were independent.

When expected cell counts were <5 in more than 20% of cells, a situation where the chi-square approximation is unreliable, we instead applied Fisher’s exact test, which computes the exact probability of observing the distribution under the null hypothesis.

For binary comparisons (e.g., malignant vs. benign by sex, or by period), we calculated odds ratios (ORs) with 95% confidence intervals (CIs) to provide an effect size estimate. Odds ratios are particularly useful for clinical interpretation because they quantify how much more (or less) likely an outcome is in one group compared with another.

For categorical variables with more than two levels (e.g., age group stratified into <40, 40–59, and ≥60 years; or laterality with right, left, bilateral), results are reported using χ^2^ test statistics, supplemented with narrative interpretation of which groups contributed most to the observed pattern.

For continuous variables, age at diagnosis was analyzed in more detail. First, we tested whether age followed a normal distribution using the Shapiro–Wilk test for normality. The test returned a significant result (*p* < 0.05), indicating that the age distribution deviated from Gaussian form and was right-skewed, with more patients concentrated in older age brackets. Because the assumptions of parametric tests (e.g., Student’s *t*-test) were not met, we used the non-parametric Mann–Whitney U test for two-group comparisons of age distributions (e.g., malignant vs. benign cases, or pre-pandemic vs. pandemic/post-pandemic). This test is based on ranking values and does not assume normality, making it appropriate for skewed clinical data.

All analyses were performed on the analyzable cohort (n = 186), which excluded 21 indeterminate cases due to lack of viable tissue or inconclusive histopathology.

#### 3.5.1. Period (2016–2019 vs. 2020–2023) Paired with Malignancy

To test whether the proportion of malignant diagnoses changed between the pre-pandemic (2016–2019) and pandemic/post-pandemic (2020–2023) periods, we applied a chi-square test of independence.

The chi-square statistic (χ^2^(1) ≈ 2.1–2.4, *p* ≈ 0.12–0.15) showed no statistically significant association between period and malignancy proportion.

We also computed the odds ratio (OR ≈ 2.1, 95% CI 0.79–5.66), which indicated that patients in the pre-pandemic years had approximately twice the odds of a malignant diagnosis compared to those in the pandemic/post-pandemic years. However, the confidence interval crossed 1.0, and the *p*-value exceeded the 0.05 threshold, confirming statistical non-significance.

Although the absolute number of parotid surgeries declined dramatically during the pandemic (Section 3.2), the relative proportion of malignancies remained stable. This suggests that oncologic and high-priority cases were maintained as surgical priorities, while benign and elective cases were disproportionately deferred. The non-significant statistical result emphasizes that, in this cohort, pandemic-related disruptions primarily affected surgical volume rather than altering the malignant-to-benign case ratio.

#### 3.5.2. Sex Paired with Malignancy

We next examined the relationship between sex and diagnosis type (benign vs. malignant tumors). Among the 157 analyzable cases, benign tumors were more frequent in females (72/126; 57.1%), while malignant tumors were relatively more common in males (19/31; 61.3%). A chi-square (χ^2^) test of independence did not show a statistically significant association between sex and diagnosis type (χ^2^(1) = 2.91, *p* = 0.088). The odds of malignancy in males were approximately twice those in females (OR = 2.11, 95% CI 0.89–4.98), although this result did not achieve statistical significance. Similarly, the relative risk (RR) indicated that males were about 82% more likely to present with malignancy compared with females.

From a clinical perspective, this distribution highlights a trend toward sex-based differences in tumor behavior: benign tumors predominated in females, while malignancies were more frequent in males. Although the current cohort size limited the power to detect statistical significance, this pattern is consistent with prior epidemiological studies reporting a slight male predominance in parotid malignancies and a higher frequency of benign lesions in females.

#### 3.5.3. Age Group (<40, 40–59, ≥60) Paired with Malignancy

Patients were stratified into three ordered age groups: <40 years (n = 32), 40–59 years (n = 70), and ≥60 years (n = 84). Across these groups, benign tumors predominated, but the proportion of malignancies increased progressively with age.

Specifically:

- In the <40 group, 22 cases were benign (68.8%) and 2 were malignant (6.3%).
- In the 40–59 group, 50 cases were benign (71.4%) and 12 were malignant (17.1%).
- In the ≥60 group, 54 cases were benign (64.3%) and 17 were malignant (20.2%).

A chi-square (χ^2^) test of independence comparing benign vs. malignant distributions across the three age groups did not reach statistical significance (χ^2^(2) ≈ 1.91, *p* ≈ 0.39), indicating that differences in malignancy rates across age strata could have occurred by chance.

To formally test for a linear age-related trend, we performed a Cochran–Armitage trend test, assigning scores 1 (<40), 2 (40–59), and 3 (≥60). The test yielded a Z ≈ 0.58 (*p* ≈ 0.56), confirming no statistically significant monotonic increase in malignancy with advancing age in this cohort.

For clinical interpretability, we collapsed age into two categories: <60 years (n = 102) vs. ≥60 years (n = 84). The odds ratio (OR) for malignancy in the ≥60 group compared with younger patients was 1.20 (95% CI: 0.55–2.63), suggesting only a modest and non-significant increased risk in older patients.

While raw numbers suggest that malignancies accumulate with age, particularly in the ≥60 years group, the statistical analysis indicates that the difference is not robust. Benign tumors remained the majority diagnosis in all age strata, though the type varied: pleomorphic adenoma dominated in younger patients, while Warthin tumor was the most common benign lesion in older adults.

#### 3.5.4. Laterality (Right, Left, Bilateral) Paired with Malignancy

Laterality distribution was examined for the analyzable cohort (n = 186). Overall, right-sided lesions were slightly more common (n = 96; 51.6%) than left-sided lesions (n = 87; 46.8%), with bilateral involvement rare (n = 3; 1.6%).

When stratified by diagnosis type:

- Right-sided lesions: 78 benign (81.3%) and 18 malignant (18.7%).
- Left-sided lesions: 74 benign (85.1%) and 13 malignant (14.9%).
- Bilateral lesions: 2 benign (66.7%) and 1 malignant (33.3%).

A chi-square (χ^2^) test of independence was applied to evaluate the relationship between laterality (right, left, bilateral) and malignancy status. The test result was χ^2^(2) ≈ 0.06, *p* = 0.97, indicating no statistically significant association between side of involvement and likelihood of malignancy.

For binary comparison of right vs. left lesions only, the odds ratio (OR) for malignancy in right-sided lesions compared with left-sided lesions was 1.31 (95% CI: 0.59–2.95). This suggests a slightly higher risk for right-sided malignancies, but the wide confidence interval and non-significant *p*-value confirm that this difference is likely due to chance.

Malignant tumors were nearly evenly distributed across right and left parotid glands, with one exceptional bilateral malignant case. Benign tumors predominated across all laterality categories. These findings, consistent with prior reports, confirm that laterality is not a clinically relevant predictor of tumor behavior in parotid neoplasms.

#### 3.5.5. Age as a Continuous Variable

Age was analyzed as a continuous variable to explore potential differences between clinical subgroups. The Shapiro–Wilk test for normality indicated that the age distribution deviated significantly from a Gaussian pattern (*p* < 0.05), with a right-skewed shape and a median value lower than the mean. This skewness was consistent with a subset of very elderly patients, resulting in a longer upper tail of the distribution. Given the non-normal distribution, non-parametric methods were selected for group comparisons.

Mann–Whitney U tests were applied to compare median ages between (i) malignant and benign cases, and (ii) cases diagnosed in the pre-pandemic period (2016–2019) versus the pandemic/post-pandemic period (2020–2023). In both comparisons, no statistically significant differences were detected (*p* > 0.05). The overlap of interquartile ranges and the absence of a meaningful shift in medians suggest that age was not a major determinant of malignancy status in this cohort, nor did the age profile of patients change appreciably between study periods.

These findings imply that the demographic structure of patients with parotid lesions remained stable over time, and that the higher malignant case counts observed in older age groups (Section 3.5.3) reflected categorical trends rather than large shifts in the underlying continuous age distribution.

#### 3.5.6. Temporal Trends in Annual Case Volume (2016–2023)

To assess how the total number of parotid tumor cases evolved across the study period, we analyzed annual case counts from 2016 through 2023. The data showed a plateau during 2017–2019 (40–41 cases per year), followed by a sharp decline starting in 2020. The lowest point occurred in 2021, with only 11 recorded cases, after which partial recovery was noted in 2023 (21 cases). Despite this rebound, volumes remained well below pre-pandemic levels.

A simple linear regression was applied, with year as the independent variable and annual case count as the dependent variable, to test whether there was a systematic temporal trend. The model produced a slope of –3.51 cases/year (SE = 1.83), with R^2^ = 0.44 and *p* ≈ 0.074. Although the *p*-value did not reach the conventional 0.05 threshold for statistical significance, the negative slope reflects a clinically relevant average decline of 3–4 cases per year.

**Figure 11:**
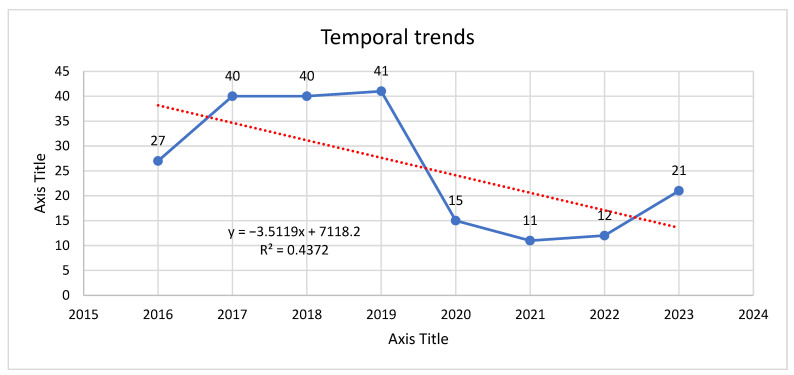
Annual case volume of parotid tumors between 2016 and 2023. A negative linear trend was observed (slope = −3.51 cases/year, *p* = 0.074), reflecting an overall decline in case counts with a sharp drop during the COVID-19 pandemic.

Visual inspection of the regression line (Figure 11) highlights that the decline was not gradual but rather characterized by an abrupt step decrease coinciding with the COVID-19 pandemic years (2020–2022). The shaded interval in the plot corresponds to this period of restricted elective surgical activity and reduced diagnostic throughput, which disproportionately affected benign and indeterminate lesions.

From a healthcare systems perspective, these findings suggest that the fall in surgical case volume was primarily driven by external constraints (pandemic-related service reductions) rather than a true epidemiological change in disease incidence. The partial rebound observed in 2023 may represent deferred surgical cases or improved patient access after restrictions were lifted. Ongoing surveillance will be necessary to determine whether volumes eventually return to pre-2020 levels or stabilize at a new baseline.

### 3.6. Key Findings

This study analyzed 207 consecutive parotid nodular lesion cases diagnosed between 2016 and 2023, of which 186 were histopathologically analyzable. The findings integrate demographic patterns, temporal dynamics, histopathological profiles, malignancy characteristics, and statistical associations, which are summarized in Table 8 presented below.

Collectively, these findings underscore the stability of malignancy incidence across demographic and temporal strata despite pronounced fluctuations in total case volume. The data suggest that surgical prioritization preserved malignant case detection during pandemic constraints, while benign and indeterminate cases were disproportionately deferred.

## 4. Discussion

In the first year of pandemic, 2020, 53,583 new cases of salivary gland malignant neoplasm were detected globally, with 22,778 recorded fatalities from this cause [9,23]. It is already established through many scientific papers that the predominant malignant types of parotid gland tumors include mucoepidermoid carcinoma (30%), adenoid cystic carcinoma, and malignant mixed tumours [24].

Quite frequently, the delays in diagnosis are due to the slow growth and lack of symptoms of this type of tumors, resulting in later stages of the disease, which have a direct impact on prognosis and therapeutic management and indicate worse survival rates. Early diagnosis of precancerous lesions or early-stage malignancies is therefore essential since it raises the chance of a cure and dramatically lowers the rates of death and morbidity [9,25,26]. The COVID-19 pandemic’s isolation and social distancing measures had a negative impact on the health system and the early identification and diagnosis of multiple cancer types, leading to a significant drop in screening, appointments, treatments, and surgeries [9].

### 4.1. Global Trends in Parotid Tumor Pathology During the Pandemic

An interesting study examining a potential delay in parotid tumor detection brought on by the COVID-19 pandemic in Brazil, is that of Leite et al. [9], who conducted a longitudinal, and analytical study regarding salivary gland cancer incidence from 2019 until 2022. Their analysis revealed differences between the years before and after the epidemic, but there were no statistically significant differences between the periods examined. The total number of tumors decreased from 2019 to 2020, with an increase in 2021, probably due to the restricted health system functioning during pandemic. For every studied year, malignant parotid neoplasia continued to be the most common, especially in males, in the 5th and 6th decade of life and the most frequent treatment modality remained the surgical one, followed by radiotherapy. When comparing to a study from The Netherlands [27], a similar scenario is described: there was a noticeable decline in the occurrence of head and neck cancer, specifically oral and laryngeal carcinoma, during the initial COVID-19 outbreak in 2020, with rates being almost 25% lower compared to 2018 and 2019. Nevertheless, after healthcare services returned to normal, the second (2021) and third (2022) waves of the pandemic suggested a possible rebound, as evidenced by increased registrations of such cases. Consequently, it can be stated that 2022 signified the start of addressing and managing the impacts of underdiagnosis and underreporting. In a similar vein, when examining oral cancer, Lo Giudice et al. [28] observed a minor reduction in the average number of cases in 2020 compared to 2019, categorizing this change as negligible, thus indicating that there was no increase in treatment delays during the COVID-19 pandemic in Italy. Our analysis revealed a similar temporal trend: a plateau between 2017 and 2019, followed by a sharp decline from 2020 to 2022 and a partial rebound in 2023.

Several studies which investigated the relation between Coronavirus disease 2019 and salivary glands pathology concluded that, although viral loads of the coronavirus may be present in saliva samples consistently [29], there are rarely salivary gland symptoms present. Guarino et al. [30] conducted a systematic evaluation of clinical and diagnostic features for SARS-CoV-2-infected patients with parotitis and/or submandibular/sublingual sialadenitis. After applying the inclusion/exclusion criteria, they analysed 10 manuscripts out of 166, totaling 27 patients with parotid gland sialadenitis, from January 2020 to February 2024. The paper concluded that COVID-19-related sialadenitis in SARS-CoV-2 patients is rare.

Another group of researchers from Brazil conducted postmortem biopsies on 24 COVID-19 cases demonstrated that the salivary glands act as a reservoir for SARS-CoV-2, with viral replication serving as an effective mechanism of dissemination through infected saliva droplets [29].

Regarding the multifactorial development of salivary gland neoplasms, genetic and environmental factors are known to play a role and although direct connections have not yet been proven, ionising radiation, chemical exposure, obesity, autoimmune diseases, and viral infections (Epstein–Barr virus in lymphoepithelial carcinoma) have all been suggested. Both benign and malignant salivary gland neoplasms can arise as a result of chromosomal translocations and mutations in tumor suppressors/oncogenes [31].

In the United States, salivary gland cancers affect around 1.1 out of 100,000 people [31,32], with the most frequent three histological forms being: mucoepidermoid carcinoma, adenoid cystic carcinoma and acinic cell carcinoma.

A 2018–2022 Italian study evaluated primary and secondary malignancies and the clinical features and prognosis of malignant parotid epithelial tumors. They determined that mucoepidermoid carcinoma was the most common and the malignant parotid carcinoma prognoses depend on tumor stage, histological type, grade, facial nerve paralysis, migration beyond the gland, and cervical lymph node involvement. Cutaneous squamous cell carcinoma in the head and neck causes most parotid gland metastases. Regarding treatment options, surgery is the main treatment for removable tumors. If there are negative pathology signs, such as high-grade tumors, perineural or vascular invasion, near or positive margins, or advanced staging, adjuvant radiation or chemo-radiotherapy should be explored. Radiotherapy and chemo-radiotherapy are the only treatments for non-surgical tumors [33].

Andrianopoulou et al. compared malignant parotid tumour diagnosis and treatment from 2010–2018 to earlier research [34]. Primary epithelial malignant parotid tumours were found in 45 of 104 patients between 2010 and 2018. Most cases (22.2%) were mucoepidermoid and squamous cell carcinoma. The average age was 61 and males outnumbered females 3:2. Like previous studies, ultrasonography (50%) MRI (80%) and intraoperative frozen sections (88.9%) were employed for diagnosis [35,36]. TNM staging showed T1 as the most common stage (46.7%) and lymph node metastasis in 31.1%, notably in high-grade and T3 tumours. Full or partial parotidectomy produced facial nerve palsy, seromas, and salivary fistulas. In 67% of instances, neck dissection revealed 20% concealed lymph node metastases. 56% of patients had tumor-free resection margins, while 10 had disease recurrence. Low-risk high-grade tumour patients may have hidden lymph node metastases. The 2016 and 2020 guidelines propose elective neck dissection for high-grade adenocarcinomas, squamous cell carcinomas, and T3/T4 tumours [34,37,38]. German guidelines were updated in May 2024 [39]. In accordance, high-grade tumours, T3/T4 stage, lymph node metastases, positive resection margins, and perineural invasion were considered while administering postoperative radiation therapy.

A retrospective study of 46 parotid neoplasm patients who underwent parotidectomy at the National Cancer Institute (NCI) Egypt in 2019 [40]. Patient investigations included CT scan (80.4%), ultrasonography (10.9%), or MRI (8.7%). The study showed that preoperative radiological evaluation might detect malignant patients and alarming cervical nodes, supporting the overall results. CT scans were used for most radiological preoperative examinations, producing the best statistical results.

Another study conducted from 2019 to 2022 included 32 total cases of salivary gland tumors among individuals aged 11 to 79 years, with a mean age of 45.5 years [41]. Male patients had more malignant lesions, whereas females had more benign ones. Histopathological examination showed that benign tumours were mostly pleomorphic adenomas (59.4%) and malignant tumours were mucoepidermoid carcinomas (40.6%), confirming previous research [42,43]. In 154 parotid tumour patients, Maahs et al. [44] discovered that pleomorphic adenoma was the most common benign tumour while mucoepidermoid carcinoma was the most common malignant tumour. The facial nerve was preserved with superficial parotidectomy, the most usual surgery. Reversible facial nerve branch paresis was most prevalent. Benign tumours had a 1:2 male-female ratio, while malignant tumours had 1:1. The median age at presentation was 45 years for benign tumours and 59 years for malignant tumours.

The age and sex trends in similar studies align with those observed in our research, as does the prevalence of malignant tumours. Most comparable studies indicate the prevalence of adenocarcinoma or mucoepidermoid carcinoma. The observed difference in our study arises from the study design: the admission codes pertained to nodular tumours, which were the focus of the research, resulting in the majority being confirmed as benign during pathological examination. However, there were a few exceptions: patients who, despite being admitted with nodular parotid tumour formations, were found upon pathological examination to exhibit characteristics of malignancy, specifically squamous cell carcinoma.

In another study conducted from 2019 to 2022, 32 salivary gland tumour cases were reported in people aged 11 to 79, with a mean age of 45.5 years [41]. Male patients had more malignant lesions, whereas females had more benign ones. Histopathological examination showed that benign tumours were mostly pleomorphic adenomas (59.4%) and malignant tumours were mucoepidermoid carcinomas (40.6%), confirming previous research [42,43]. In 154 parotid tumour patients, Maahs et al. [44] discovered that pleomorphic adenoma was the most common benign tumour while mucoepidermoid carcinoma was the most common malignant tumour. The facial nerve was preserved with superficial parotidectomy, the most usual surgery. Reversible facial nerve branch paresis was most prevalent. Benign tumours had a 1:2 male-female ratio, while malignant tumours had 1:1. The median age at presentation was 45 years for benign tumours and 59 years for malignant tumours.

In 2022, an international group of researchers conducted a multicenter cohort analysis study of salivary gland tumour data from centers worldwide. These findings aimed to establish a foundation for future research on the epidemiological geography of these malignancies. The analysed data encompassed age, gender, location, and histological diagnosis from fifteen centres representing the majority of the World Health Organisation (WHO) geographical regions between 2006 and 2019, aiming to address the limitations of most epidemiological studies on salivary gland tumours for various reasons. The study included 5739 patients, 65% benign and 35% malignant. Small female propensity (54%), peak occurrence between the fourth and seventh decades for benign and malignant tumours. 68% of salivary gland tumours (SGT) were in the large glands and 32% in the minor glands. The parotid gland hosted 70% of benign tumours, with Warthin’s tumour (17%) and pleomorphic adenoma (70%) being the most common benign tumours, while mucoepidermoid carcinoma (26%) and adenoid cystic carcinoma (17%) were the most common malignant cancers [45].

Regarding the mortality rate and prognostic factors in malignant parotid tumour development, several studies have reported a reduced 15-year overall survival rate, ranging from 39.8% to 42% [46,47]. A retrospective study involving 193 patients investigated prognostic markers and overall survival over a 15-year follow-up period for individuals with malignant salivary gland tumours, indicating an overall survival rate of 67.4% and a survival duration of 116 ± 6 months. Their findings indicated that sex affects case prognosis, with males exhibiting a higher mortality rate. The patient’s origin influences outcomes, with individuals from rural areas demonstrating a poorer prognosis than those from urban districts. The advanced clinical stage, according to the TNM classification, is recognised as a significant prognostic factor. The T and M stages significantly influenced mortality rates and patient survival, aligning with existing data, particularly indicating an unfavourable prognosis in stage IV cases. Patients with inadequate surgical margins demonstrated reduced survival rates. Additionally, the analysis revealed that chemotherapy was the only significant predictor, with patients receiving chemotherapy demonstrating decreased survival rates. Malignant salivary gland tumours exhibit a restricted response to conventional chemotherapy, often being allocated to palliative care. The multivariate analysis of patient age distribution indicated that only education level presented an independent association with patient prognosis [48].

### 4.2. Limitations of the Study and Future Perspectives

Limitations of our study derive from its retrospective nature, namely the lack of complete information regarding surgical management, possible complications and postoperative outcomes. Further studies will be necessary to follow the evolution of cases in detail, especially those of a malignant nature.

It appears that the preponderance of benign tumour cases reported in our study is corroborated by comparable studies conducted in other regions of the world over comparable amounts of time. The results of our study regarding malignant tumours do not encompass the entire malignant pathology; rather, they pertain exclusively to cases that were initially diagnosed as nodular tumours and subsequently diagnosed as oncological cases through anatomopathological analysis. Studies that concentrate on the pathology of malignant tumours in the parotid gland are necessary to obtain a comprehensive understanding of malignant epidemiology, especially knowing that early diagnosis through improved technologies is critical for achieving tumor-free margins during surgery and a good prognosis of these cases. The principal objective of this study was to examine benign parotid nodal tumors, with a hypothesised variance between the pre- and post-pandemic periods.

Salivary gland neoplasms represent a heterogeneous and complex category of tumours that are optimally managed in specialised centres. Even the most prevalent benign neoplasms, particularly pleomorphic adenoma, require meticulous surgical management and subsequent monitoring. The morphological diversity observed both between and within tumour types necessitates that careful histological examination of an excised specimen remains the gold standard for diagnosis. Diagnosing small incisional biopsies can be challenging, particularly for clear cell tumours and in distinguishing among polymorphous adenocarcinoma, pleomorphic adenoma, and adenoid cystic carcinoma. In 2002, Speight et al. emphasized the importance of the 4 cm rule, that has demonstrated utility as a clinical guide for behaviour and outcomes. Stage is likely more significant than grade when evaluating prognosis and the size of the tumour at presentation is a significant predictor of prognosis [49]. When considering this type of pathology, the prognostic factors and clinical outcome in parotid gland tumours are crucial considerations. The most prevalent significant prognostic factors for parotid gland malignancy, according to extensive research in the literature, are age, sex, T stage, N stage, grade, and perineural invasion. Gender, age, T stage, N stage, tumour grade, lymphovasculer invasion, perineural invasion, extracapsular extension, surgical margin, radiation dose, and distant metastases were predictive variables associated with overall survival, according to a Turkish study [50]. Through precise preoperative evaluation, which includes a comprehensive history taking, clinical examination, and investigations including MRI, CT scan, and fine-needle aspiration cytology, recurrent malignant salivary glands and their consequences can be prevented. This approach ensures a precise diagnosis and enables the selection of the best surgical and adjuvant therapies when necessary [51,52].

During the first two decades of the 21st century, humanity has encountered several pandemics, including SARS-CoV-1, the 2009 H1N1 influenza, chikungunya in 2013, and SARS-CoV-2, as well as various epidemics (cholera, measles, yellow fever, Zika, MERS, etc.). The characteristics of pathogens in outbreaks present significant challenges to public health systems, particularly in low- and middle-income countries. During outbreaks, epidemics, and pandemics, the global community anticipates the World Health Organisation to assume a leadership role and offer solutions to the prevailing crisis. Since its establishment in 1948, the WHO has assumed leadership roles in significant global medical emergencies. In significant crises, the World Health Organisation must engage and exchange strategies with comparable entities such as the Centres for Disease Control and Prevention, the Coalition for Epidemic Preparedness Innovations, and the Global Research Collaboration for Infectious Disease Preparedness. Outbreaks and acute public health risks are often unpredictable and require targeted strategies. In 2005, the International Health Regulations (IHR) established a fundamental legal framework delineating the rights and obligations of countries in addressing public health incidents and emergencies that may transcend international boundaries. The International Health Regulations (IHR) are legally binding for 196 countries, which encompasses the 194 Member States of the World Health Organisation (WHO). Effective management during an outbreak necessitates the organisation of trained personnel possessing expertise in clinical management, public health, epidemiology, and various medical specialities. Countries that perform well during outbreaks typically possess strong preventive public health systems alongside effective curative health services. During an outbreak, absolute numbers provide opportunities for research and can yield significant results in diagnostics and therapeutics. Substantial information can be gathered prospectively in observational studies to obtain significant data. Finally, robust infection control measures must be implemented among healthcare workers and the community as well [53].

## 5. Conclusions

In summary, this study demonstrates that despite the notable reduction in surgical case volume during the COVID-19 pandemic, the relative distribution of parotid tumor pathology remained consistent. Malignant lesions continued to present predominantly in older patients and in males, without statistically significant differences across demographic or clinical subgroups. The maintenance of stable malignancy detection rates, even under restricted surgical capacity, highlights the effectiveness of oncologic prioritization during a period of healthcare disruption. Continued longitudinal monitoring will be essential to determine whether the observed post-pandemic recovery represents a return to baseline patterns or a shift in long-term clinical practice.

## Figures and Tables

**Table 1:** Age group distribution.

Age Group	Count	Percent (%)
60+	99	47.8
40–59	76	36.7
<40	32	15.5

**Table 2:** Comparison of case numbers and proportions between the pre-pandemic group (2016–2019) and during and post pandemic group (2020–2023).

Age Band	F	M	2016–2019	2020–2023
<40	17	15	21	11
40–59	42	34	57	19
60+	43	56	70	29

**Table 3:** Histopathological diagnoses.

Histopathological Diagnosis	2016–2019	2020–2023	Total
Pleomorphic adenoma	42	22	64
Warthin tumor	36	23	59
Adenocarcinoma (salivary)	4	1	5
Branchial cyst (intraparotid)	4	0	4
Mucoepidermoid carcinoma	6	0	6
Squamous cell carcinoma	4	2	6
Non-Hodgkin lymphoma	3	0	3
Basal cell carcinoma	2	0	2
Chronic sialadenitis	5	2	7
Lipoma	2	0	2
Lymphoma (other than Hodgkin)	2	1	3
Myoepithelial carcinoma	2	0	2
Reactive lymph node	2	0	4
Salivary duct cyst	2	0	2
Acinic cell carcinoma	1	1	2
Acute suppurative sialadenitis	1	0	1
Benign lymphoepithelial lesion	1	0	1
High-grade mucoepithelioid carcinoma (G3)	1	0	1
Salivary cyst	1	0	1
Metastatic melanoma to lymph node/parotid region	1	0	1
Epidermoid cyst	1	0	1
Oncocytic carcinoma	1	0	1
Sarcoma (pleomorphic)	1	0	1
Cribriform adenocarcinoma of salivary gland	0	1	1
Desmoid-type fibromatosis	0	1	1
Normal adipose tissue	0	1	1
Oncocytic cyst	0	1	1

**Table 4:** Malignancy by period.

Period	Benign	Malignant	Lymphoid	Non-Neoplastic	Other
2016–2019	80	26	5	18	1
2020–2023	46	5	1	4	0
total	126	31	6	22	1

**Table 5:** Malignancy by age group.

Age Band	Benign	Malignant	Lymphoid	Non-Neoplastic	Other	Total
<40	22	2	1	7	0	32
40–59	50	12	2	6	0	70
60+	54	17	3	9	1	84

**Table 6:** The malignant spectrum by diagnosis, sex, and age group.

Diagnosis	F	M	<40	40–59	60+	Total Cases
Squamous cell carcinoma	0	6	0	2	4	6
Adenocarcinoma (salivary)	3	2	0	1	4	5
Malignant epithelial carcinoma (NOS)	1	3	0	2	2	4
Mucoepidermoid carcinoma	2	4	0	2	4	6
Acinic cell carcinoma	2	0	0	0	2	2
Myoepithelial carcinoma	1	1	0	0	2	2
Basal cell adenocarcinoma	0	2	0	1	1	2
Sarcoma (pleomorphic)	1	0	0	0	1	1
Lymphoma (all subtypes)	4	2	2	1	3	6
Metastatic melanoma to parotid LN	1	0	0	1	0	1
Oncocytic carcinoma	0	1	0	0	1	1
Cribriform adenocarcinoma of salivary gland	1	0	0	0	1	1
Total	12	19	2	10	19	31

**Table 7:** Spectrum of benign parotid tumors by diagnosis, sex, and age group.

Diagnosis	F	M	<40	40–59	60+	Total Cases
Pleomorphic adenoma	38	26	20	27	17	64
Warthin tumor	32	27	2	21	36	59
Lipoma	1	1	0	0	2	2
Oncocytic cyst	1	0	0	0	1	1
Total	72	54	22	48	56	126

**Table 8:** Summary of Key Findings (2016–2023 Cohort).

Domain	Key Findings
Sample Size & Demographics	n = 207 total, 186 analyzable; 105 males, 102 females; Mean age 56.5 (median 59; IQR 46–68); Age groups: <40 (15.5%), 40–59 (36.7%), ≥60 (47.8%)
Temporal Trends	Plateau 2017–2019 (40–41 cases/year), sharp decline 2020–2022 (nadir 11 in 2021), partial rebound 2023 (21 cases); Malignancy proportion stable between periods
Histopathology	Benign tumors: 60.9% (Pleomorphic adenoma n = 64, Warthin tumor n = 59); Malignant tumors: 15.0% (n = 31; SCC n = 6, MEC n = 6, Adenocarcinoma n = 5, Lymphoma n = 6); Other categories: non-neoplastic n = 22, stromal rare n = 1
Malignancy Patterns	Malignancy prevalence: <40 (6.3%), 40–59 (17.1%), ≥60 (20.2%); Higher in males (20.2%) vs. females (13.0%); No lateral predilection
Statistical Analysis	No significant differences by sex, age group, laterality, or period (all *p* > 0.05); OR: ↑ risk in males (2.1) and ≥60 years (1.2), NS; Linear regression: –3.5 cases/year, *p* = 0.074

## Data Availability

The data will be available from the corresponding authors upon reasonable request.

## Abbreviations

The following abbreviations are used in this manuscript:

SGT	Salivary gland tumours
WHO	World Health Organization
EHR	Electronic Health Record
FNA	Fine needle aspiration
IHR	International Health Regulations
